# Biomechanical Analysis of Injury Risk in Two High-Altitude Landing Positions Using Xsens Inertial Units and EMG Sensors

**DOI:** 10.3390/s24216822

**Published:** 2024-10-24

**Authors:** Xuewu Yao, Haojie Li, Chen Xiu

**Affiliations:** School of Athletic Performance, Shanghai University of Sport, Shanghai 200438, China; xuewusus@163.com (X.Y.); 202121070037@mail.bnu.edu.cn (H.L.)

**Keywords:** biomechanics, landing techniques, joint forces, muscle activation, injury risk

## Abstract

High-altitude landing maneuvers can pose a significant injury risk, particularly when performed with different landing techniques. This study aims to compare the biomechanical parameters and injury risks associated with two landing positions—staggered foot landing and simultaneous bilateral landing—using Xsens inertial units and electromyography (EMG) sensors. A total of 26 university students (13 males, 13 females) participated in this study. Kinematic data were collected using inertial measurement units (IMUs), muscle activity was recorded with EMG, and ground reaction forces were captured using 3D force plates. The data were processed and analyzed using the AnyBody modeling system to simulate joint forces, moments, and muscle activation. This study found that simultaneous bilateral landing exhibited greater hip flexion-extension, knee flexion-extension, and ankle inversion. Vertical joint forces were also significantly higher in the hip, knee, and ankle during simultaneous bilateral landing. Staggered foot landing showed higher muscle forces in the gluteus maximus, iliopsoas, and quadriceps femoris (*p* < 0.001). The EMG analysis revealed significant differences in the biceps femoris (*p* = 0.008) and quadriceps femoris (*p* < 0.001). These findings suggest that simultaneous bilateral landing increases joint load, while staggered foot landing increases muscle activation, which may lead to different injury risks between the two techniques.

## 1. Introduction

In sports and daily activities, the landing maneuver is a common and important motor gesture, which usually occurs in a variety of contexts such as jumping, crossing, and landing [1,2]. Landing is not only a key aspect of athletic performance but also closely related to injury risk [3]. For specific groups of people, such as army soldiers landing quickly during obstacle training, professional athletes jumping frequently during high-intensity training, or even ordinary people performing high-altitude jumps, the biomechanical properties of landing can affect their injury probability [4]. Previous studies have shown that the mismatch between the impact force generated during landing and the carrying capacity of the lower limbs is one of the main causes of injury [5]. Barber-Westin et al. analyzed the landing maneuvers of athletes and found that the load on the knee joint at the moment of landing can be up to five times of the body weight, which significantly increases the risk of injury [6]. In addition, Gheidi conducted a study on high altitude landing in military training and pointed out that incorrect landing postures, such as failure to properly flex the knee, are prone to ligament injuries and soft tissue injuries [7]. Larsson, in his observation of professional athletes, found that there was a significant correlation between frequent high-intensity landing training and knee injuries [8]. And Losciale’s study showed that the excessive impact force during landing directly led to joint degradation and injury. Therefore, it is particularly important to study the biomechanical characteristics of the landing posture in depth in order to explore how to reduce sports injuries [9].

Daily landing postures can be broadly categorized into two types: two-foot simultaneous landing and two-foot anterior-posterior landing, which have significant differences in biomechanical properties and injury risk [10]. According to previous studies, when landing on both feet at the same time, the center of gravity of the body cannot be effectively dispersed, resulting in a significant increase in the impact force on the joints, thus increasing the risk of injury [11]. In their survey, respondents’ feedback showed that many athletes tend to use the way of landing on both feet at the same time when landing at a height, but the potential risk of this way has not been paid enough attention [12]. Although existing research suggests a higher risk of landing on both feet at the same time, relevant scientific quantitative studies are still insufficient. This research gap makes an in-depth exploration of how different landing postures affect injury risk an important current research direction in the field of sports biomechanics.

In order to realize the in-depth analysis of high landing postures, Xsens inertial sensors and surface electromyography (EMG) sensors were selected in this study. Xsens inertial sensors are high-precision motion capture devices that can record the three-dimensional acceleration, angular velocity, and displacement data of an athlete in real time during the landing process [13]. The advantage of this technology is its seamless integration and high reliability, which can provide accurate data support for biomechanical analysis. Combined with surface electromyography, researchers are able to monitor the electrical activity of the muscles involved during landing and assess their activation patterns and intensity. This combination of analytical methods provides an effective means of understanding the load distribution of the muscles during different landing postures. In addition, this study utilized the Xsens inertial sensor and AnyBody musculoskeletal simulation system to build a musculoskeletal model of high place landing, which enabled the detailed analysis of joint and muscle forces under different postures. This novel analysis method will help us to understand the biomechanical mechanisms of the landing maneuver more comprehensively and fill the gap in the current research.

The innovation of this study lies in its integrated use of advanced Xsens inertial sensors and surface electromyography to delve into the effects of high landing postures on injury risk. By analyzing the biomechanics of different landing postures, this study not only provides scientific training guidance for athletes and coaches to reduce the risk of injuries but also helps to promote research progress in the field of sports biomechanics. In addition, the results of this study will provide theoretical support for the safety of sports training, which is of great significance for improving the training efficiency and competitive performance of athletes. Through this study, we expect to establish a systematic assessment method to help coaches and athletes identify potential risks and take appropriate preventive measures. Overall, the results of this study will have a profound impact on athletic training and its related fields, providing a valuable foundation for future research and practice

## 2. Methods

### 2.1. Participants

A total of 26 active university students (13 males, 13 females) participated in this study. The mean age of the participants was 19.82 ± 2.15 years, with an average height of 171.11 ± 3.01 cm and an average weight of 62.01 ± 4.85 kg. The sample size was calculated by GPower 3.1 to achieve 80% power with an effect size of 0.25, and this study met the minimum requirement of 23 participants. All participants had previously engaged in at least one or more jump-related sports, such as basketball, volleyball, soccer, or badminton. Individuals who were medically restricted from participating in jump tasks or who had suffered lower limb injuries within the past six months were excluded from this study. Additionally, participants with a history of vestibular disorders or connective tissue diseases were also excluded.

Written informed consent, approved by the Institutional Review Board (IRB) of Shanghai University of Sport, was obtained from all participants prior to this study. This study adhered to the ethical guidelines outlined in the Declaration of Helsinki.

### 2.2. Study Design

Participants attended a single laboratory session to complete the experiment. Upon arrival, they first engaged in a 15 min warm-up routine consisting of light jogging in place, squats, and other basic warm-up exercises. Following the warm-up, participants were introduced to the landing tasks by performing practice trials from a lower height of 15 cm. Each participant completed one to three practice trials, depending on when they felt comfortable and familiar with the task.

The experimental protocol consisted of two distinct landing tasks: staggered foot landing (one foot landing before the other) and simultaneous bilateral foot landing. Each task was performed three times. The order of the landing tasks was randomized to control for order effects.

### 2.3. Procedure

The experimental tasks consisted of two distinct landing maneuvers.

- Staggered Foot Landing

**Figure 1:**
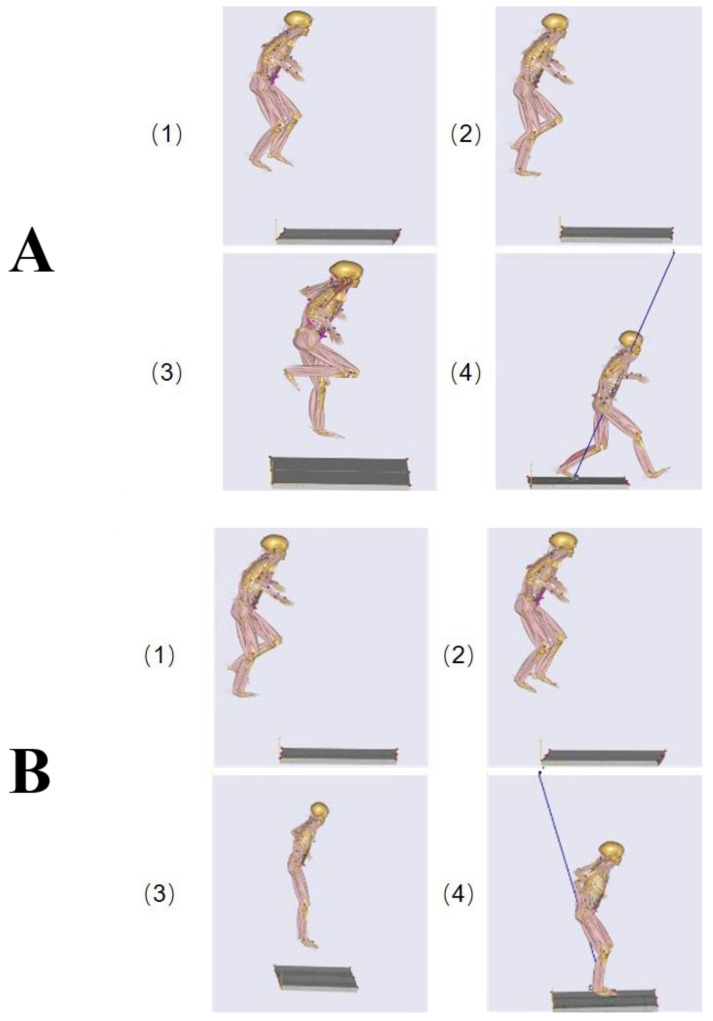
Different landing tasks: (**A**) staggered foot landing; (**B**) simultaneous bilateral foot landing.

Participants stood on a platform 120 cm above a force plate, with their feet shoulder-width apart. They were instructed to jump forward and aim for the center of the force plate, ensuring that one foot landed first, followed immediately by the other. The goal was to achieve a staggered landing pattern (Figure 1A).

- Simultaneous Bilateral Foot Landing

In this task, participants again stood on the 120 cm platform, with feet shoulder-width apart. They were instructed to jump forward, aiming for the center of the force plate, ensuring that both feet landed simultaneously (Figure 1B).

Prior to the experimental tasks, participants donned the IMU sensors and EMG sensors during the practice trials. The sensors were affixed using adjustable straps, and adjustments were made until the participants reported feeling comfortable performing the practice movements.

Following the setup, we provided participants with detailed demonstrations and clear guidance to standardize their landing techniques. Specifically, we instructed them on the appropriate landing areas (ensuring that the feet, not the toes or heels, made even contact with the ground) and the proper body posture (maintaining an upright torso and controlled knee flexion). Throughout the process, participants were encouraged to maintain the natural flow of their movements without compromising their individual posture. However, they were guided to follow the demonstrated landing position to ensure consistency across trials.

### 2.4. Measurements

The data collection for this study was divided into three components: inertial measurement unit (IMU) data, electromyography (EMG) recordings, and ground reaction force (GRF) measurements.

#### 2.4.1. Inertial Measurement Units (IMUs)

Following the warm-up, participants were fitted with the MVN Biomech motion capture system (Xsens, Amsterdam, Netherlands), consisting of 17 inertial sensors. These sensors were placed on various parts of the body to record linear acceleration, angular velocity, and magnetic field intensity in three dimensions. The sensors were positioned according to the manufacturer’s guidelines and placed on the feet, thighs, wrists, forearms, upper arms, chest, lumbar spine, and head and below the knees. Once the sensors were in place, calibration was performed to ensure accurate data collection throughout the testing session.

#### 2.4.2. Electromyography (EMG) Recordings

**Figure 2:**
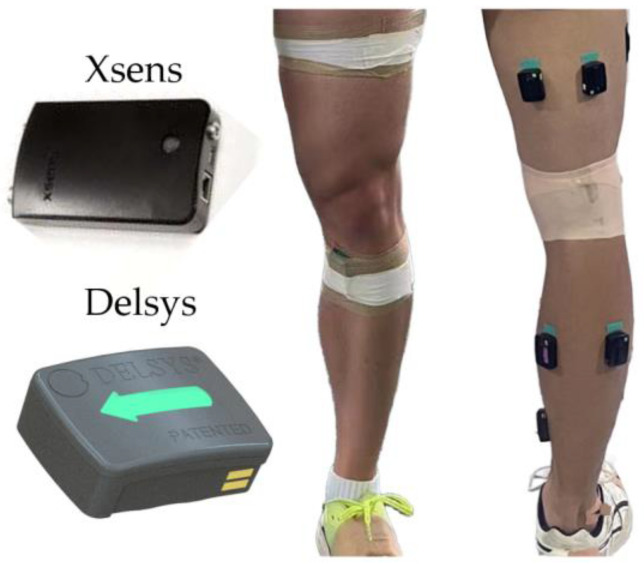
Wearing position of inertial measurement unit and electromyography sensor.

Surface electromyography (EMG) was used to measure the muscle activity of five key muscles on the dominant side of the body: the gluteus maximus, quadriceps femoris, biceps femoris, gastrocnemius, and tibialis anterior. Before attaching the electrodes, the participants’ skin was prepared by shaving and cleaning the electrode sites with alcohol to minimize impedance. EMG sensors (Delsys Inc., Boston, MA, USA; inter-electrode distance: 1 cm) were then placed on the muscle bellies following the SENIAM guidelines. The electrodes were secured with adhesive tape to ensure that they remained in place during the trials. EMG signals were recorded at a sampling frequency of 1000 Hz (Figure 2).

#### 2.4.3. Force Plate Measurements

Ground reaction forces were measured using two 3D force plates (Kistler 928E, Kistler Group, Zurich, Switzerland), operating at a sampling frequency of 1000 Hz. These force plates provided real-time measurements of the vertical, anterior-posterior, and medial-lateral components of the ground reaction forces during landing. The static calibration error for the force plates was less than 0.5%, ensuring high accuracy in capturing the dynamic forces generated during the landing tasks.

### 2.5. Data Analysis

The data collected from the electromyography (EMG) recordings, kinematic measurements, and biomechanical modeling were analyzed as follows.

#### 2.5.1. EMG Signal Processing

EMG signals were recorded at a sampling frequency of 1000 Hz using the Delsys system. Prior to analysis, the raw EMG data underwent preprocessing to ensure accuracy and reduce noise. Initially, a band-pass filter with a frequency range of 20–450 Hz was applied to remove movement artifacts and electrical noise. To further reduce interference, a notch filter was used to eliminate power line noise at 50 Hz. After filtering, the EMG signals were fully rectified to convert all negative values to positive, enhancing the representation of muscle activity.

The preprocessed EMG signals were then used to calculate two key metrics: the integrated EMG (iEMG) and the median frequency. The iEMG was used to quantify the overall muscle activation during the landing tasks, while the median frequency was used to assess muscle fatigue [14].

The Delsys EMG system also included built-in IMU sensors capable of measuring acceleration and angular velocity. During data collection, the IMU sensors were activated, and synchronization was achieved by comparing IMU data from the Delsys system with that from the Xsens system. This allowed for the precise alignment of the data and identification of movement cycles, ensuring temporal synchronization between the two systems.

#### 2.5.2. Kinematic and Biomechanical Analysis

To simulate the loads experienced by the musculoskeletal system, the AnyBody Modeling System (version 7.4) was used. The kinematic data collected by the Xsens system was exported in BVH format and served as the input for driving the full-body biomechanical model in AnyBody. The model consisted of solid body segments, each connected by joints, representing the major joints and limbs of the human body.

The analysis was divided into three key stages.

- Model Scaling

First, the biomechanical model was scaled to match the individual anthropometric measurements of each participant. This ensured that the model accurately reflected each subject’s body size and proportions, enhancing the precision of the subsequent simulations.

- Kinematic Analysis

The kinematic data were processed using a fourth-order low-pass digital filter with a cutoff frequency of 12 Hz. The kinematic variables of interest included hip flexion-extension, hip adduction-abduction, knee flexion-extension, ankle dorsiflexion-plantarflexion, and ankle inversion-eversion.

- Inverse Dynamics Analysis

Finally, an inverse dynamics analysis was conducted to calculate the internal forces and moments acting on the body during landing. This analysis involved the computation of muscle forces and joint reaction forces, providing insights into the biomechanical load experienced by the joints during both landing tasks. Special attention was given to the moments of peak ground impact, where the vertical joint reaction forces (N/BM), joint moments (Nm/BM), and muscle forces (N/BM) were calculated for each participant. The results were normalized relative to body mass (BM) to allow for meaningful comparisons across participants with different body weights.

All results were expressed in normalized values to account for inter-participant variability in body mass, thereby enabling the direct comparison of biomechanical loads between individuals.

### 2.6. Statistical Analysis

All statistical analyses were conducted using SPSS version 20.0 (IBM Corp., Armonk, NY, USA). Data are reported as mean ± standard deviation (SD). Initially, the data were tested for normality using the Shapiro–Wilk test to ensure that the assumptions of normal distribution were met. For data that followed a normal distribution, independent sample *t*-tests were performed to compare the kinematic variables, joint forces, joint moments, muscle forces, and EMG signals between the two landing conditions: staggered foot landing and simultaneous bilateral foot landing. The independent sample *t*-test was chosen because the correlation between the data from the two landing tasks was not significant. A significance level of *p* < 0.05 was used to determine statistical significance.

## 3. Results

**Figure 3:**
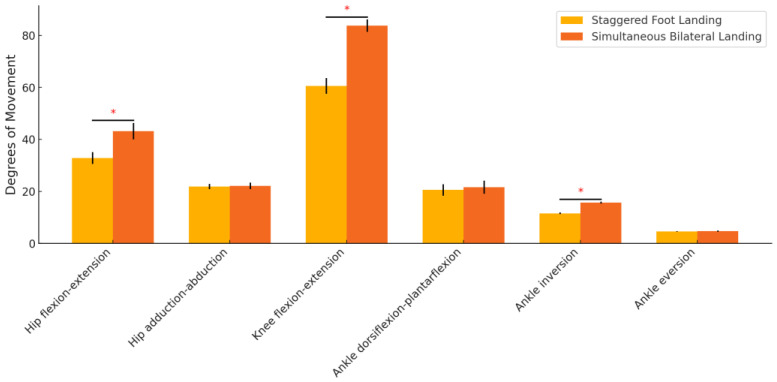
Comparison of kinematic variables between different landing positions. * indicates that the difference is significant.

The kinematic comparison showed significant differences between the two landing positions. Hip flexion-extension, knee flexion-extension, and ankle inversion all demonstrated significant differences, with higher values observed in the simultaneous bilateral landing condition (*p* < 0.001). No significant differences were found for hip adduction-abduction, ankle dorsiflexion-plantarflexion, or ankle eversion (Table 1, Figure 3).

**Figure 4:**
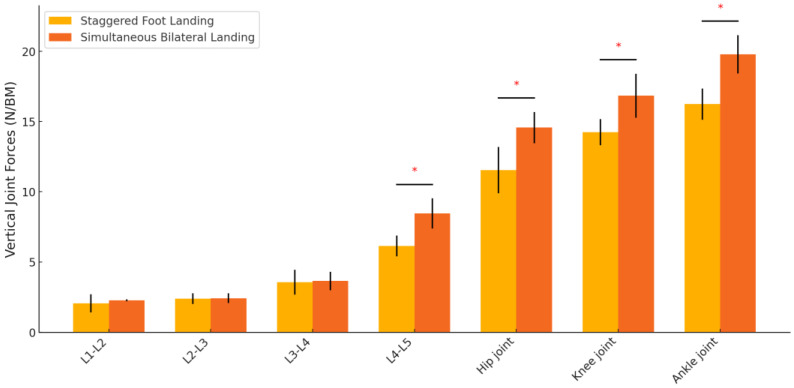
Comparison of vertical joint forces between different landing positions. * indicates that the difference is significant.

Significant differences in vertical joint forces were observed at the L4–L5 level, the hip joint, the knee joint, and the ankle joint (*p* < 0.001), with higher forces in the simultaneous bilateral landing condition. No significant differences were observed at the L1–L2, L2–L3, or L3–L4 levels (Table 2, Figure 4).

**Figure 5:**
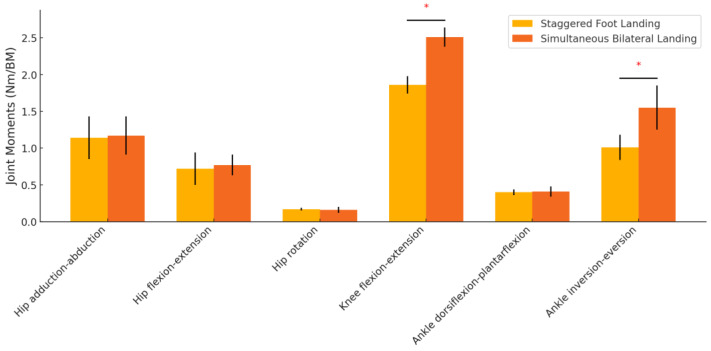
Comparison of joint moments between different landing positions. * indicates that the difference is significant.

The joint moments comparison showed significant differences in knee flexion-extension moments and ankle inversion-eversion moments (*p* < 0.001), with greater moments observed in the simultaneous bilateral landing condition. No significant differences were observed for hip adduction-abduction, hip flexion-extension, hip rotation, or ankle dorsiflexion-plantarflexion moments (Table 3, Figure 5).

**Figure 6:**
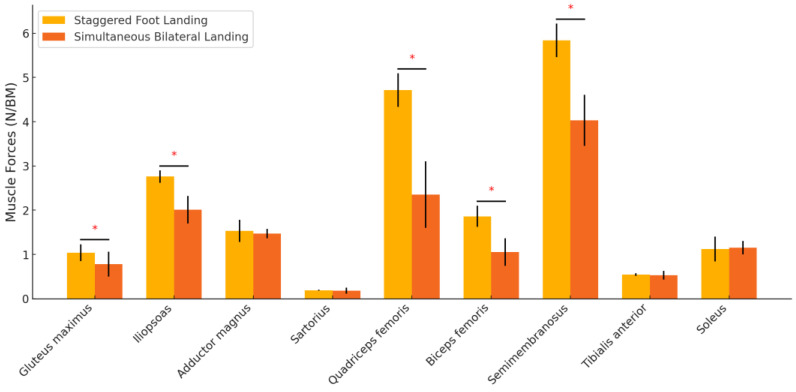
Comparison of muscle forces simulated by AnyBody for different landing positions. * indicates that the difference is significant.

Muscle forces in the gluteus maximus, iliopsoas, quadriceps femoris, biceps femoris, and semimembranosus were significantly higher in the staggered foot landing condition (*p* < 0.001). No significant differences were found in the adductor magnus, sartorius, tibialis anterior, or soleus muscles (Table 4, Figure 6).

**Figure 7:**
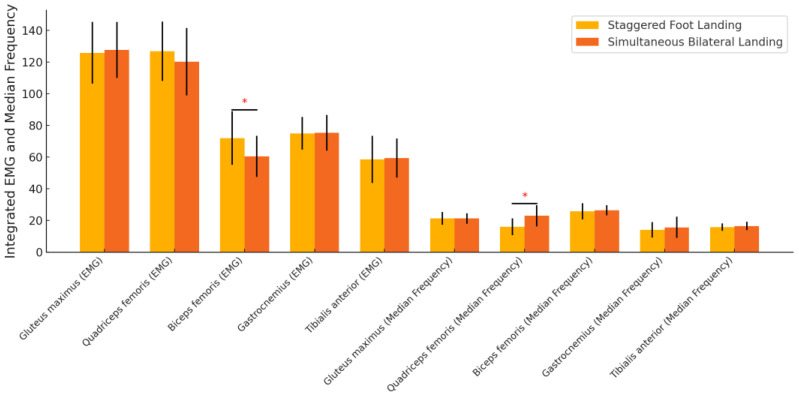
Comparison of integrated EMG and median frequency between different landing positions. * indicates that the difference is significant.

The EMG analysis revealed significant differences in the integrated EMG of the biceps femoris (*p* = 0.008) and the median frequency of the quadriceps femoris (*p* < 0.001), with lower muscle activation and higher frequency observed in the simultaneous bilateral landing condition. No significant differences were found in the gluteus maximus, gastrocnemius, or tibialis anterior muscles (Table 5, Figure 7).

## 4. Discussion

The results of this study showed that there were significant differences in kinematic parameters under different landing postures, especially under the condition of simultaneous two-foot landing, where the flexion and extension angles of the hip, knee, and ankle joints were significantly increased. This finding is consistent with previous studies by Foch, who also pointed out that simultaneous two-foot landing leads to a significant increase in joint angles and may trigger greater loading and injury risk [15]. Typically, greater joint angles mean that the joints are subjected to greater loads during landing, which may lead to reduced joint stability and increased risk of injury [3,16]. Further analysis showed that the increase in joint angle when landing on both feet at the same time may reflect the failure of individuals to effectively control their center of gravity and path of motion during landing, which may result in uneven forces on the joints [17]. Relatedly, Choffin’s study also showed that this lack of control may not only increase the risk of acute injuries, such as sprains and strains, but may also lead to chronic injuries, such as arthritis and soft tissue injuries [18]. Therefore, optimizing the landing technique, especially strengthening the awareness and control of the landing position during training, is an important strategy for injury prevention [19]. In practical application, both coaches and participants should emphasize the training of landing technique, whether it is in sports training, daily activities, or high landing situations. Simulation training is recommended to improve an individual’s adaptive ability under different landing conditions, emphasizing the use of both feet in front and back landing to reduce the burden on the joints [20]. In addition, combining strength and flexibility training and targeting the adjustment of joint angles during landing can effectively enhance joint stability [21]. This is in line with Wang’s study, which suggested that athletes’ landing ability should be improved through comprehensive training [22]. This study emphasizes the importance of the landing position for personal safety and performance, especially the potential risk when landing on both feet simultaneously. Through in-depth research, we can provide participants with more scientific training methods to ensure that they improve their performance while reducing the risk of injury.

This study found significant differences in the vertical forces on the joints in different landing positions. The forces on the L4–L5 intervertebral discs, hip joints, knee joints, and ankle joints were significantly greater under the condition of simultaneous two-foot landing. This suggests that the loads on the joints are significantly higher when landing on both feet simultaneously than when landing on both feet in a staggered manner. In contrast to previous studies, our results are consistent with Rossi’s study, which also found that landing on both feet simultaneously resulted in greater vertical loads on the joints. This consistency suggests some biomechanical generalization of simultaneous two-foot landing, suggesting that this landing pattern may lead to higher joint stresses and thus increased risk of injury [23]. However, it is worth noting that our findings differ from those of Ardakani, who did not observe a significant increase in joint stresses when landing on both feet simultaneously [3]. This may be related to the methodology of this study, the fitness level of the participants, or the specific type of exercise. Our findings emphasize the significant increase in vertical forces on the joints during simultaneous two-foot landing, especially on the hip and knee joints, which bear greater impact forces. Specifically, in the case of simultaneous landing on both feet, the joints are subjected to greater forces, which may lead to an increased risk of acute injuries (e.g., sprains and strains) and chronic injuries (e.g., arthritis and soft tissue injuries) [24]. Particularly in the hip and knee joints, higher forces mean that these areas are more susceptible to injury. In addition, excessive vertical loading may affect athletes’ performance, leading to fatigue and technical errors. In order to prevent these injuries, it is recommended that the emphasis on landing technique be reinforced in training. This approach is effective in spreading the forces during landing and reducing the direct impact on the joints [25].

Our study found that knee flexion-extension moments (*p* = 0.006) and ankle internal and external rotation moments (*p* = 0.009) were significantly greater when both feet landed simultaneously, suggesting that the joints were subjected to greater loads during this landing pattern. Compared with previous studies, our results were consistent in terms of changes in knee and ankle moments. These studies have generally pointed out that the increased joint forces in the case of simultaneous landing on both feet may lead to a greater risk of sports injuries [26,27]. This implies that optimizing the landing technique and choosing to land on both feet, front and back, may reduce the chance of injury and enhance athletic performance during practical training. However, our findings are inconsistent with some of the literature, especially with regard to the moment changes in the hip joint. Our data showed that the various moments of the hip joint (e.g., abduction, flexion, extension, and rotation) did not change significantly (*p* > 0.05), which is contrary to previous studies that suggested that hip joint forces change significantly. This may indicate that the increased loads on the knee and ankle joints during simultaneous landing of both feet are mainly due to the transient change in the center of gravity and the concentration of the impact force of landing, whereas the hip joint is able to effectively absorb part of the impact force due to its relative structural stability [28,29]. In particular, the internal and external overturning moments of the ankle joint increased significantly, and this change may be an important factor leading to ankle sprains. When landing from a high place with both feet at the same time, the concentration of the impact force and its twisting effect on the ankle joint increase the internal and external overturning moments, which increases the risk of sprains [30]. Therefore, the use of both feet to land anteriorly and posteriorly can effectively disperse the force during landing and reduce the possibility of ankle injuries.

Our study reveals significant reductions in muscle strength when landing on both feet simultaneously, particularly in key muscle groups such as the gluteus maximus, iliopsoas, quadriceps, biceps femoris, and semimembranosus. These changes reflect the body’s reduced reliance on muscle strength when landing on both feet simultaneously, which may lead to reduced control and postural balance. This reduced strength means that the body is less stable during landing, and in particular, the knee and ankle joints are subjected to a significant additional burden when impact forces are concentrated [31]. This burden not only exacerbates the risk of sports injuries but may also lead to long-term joint problems [24]. Previous research has shown that greater muscle strength helps to provide better joint control and postural stability, reducing the chance of injury. The technique of landing on both feet, fore and aft, provides better utilization of muscle strength, allowing an athlete to more effectively control body posture and distribute the impact of landing [32]. For example, the quadriceps and gluteus maximus muscles are more powerful when landing on both feet anteriorly and posteriorly, which helps to better absorb and translate the impact force and reduces the transient load on the knee and ankle joints [33]. In addition, our results showed that the indicators of muscle activation and fatigue were significantly lower when landing on both feet simultaneously than when landing on both feet fore and aft. This suggests that simultaneous landing on both feet may lead to decreased muscle activation, which in turn affects body stability and control [34]. Specifically, the lower activation level of the biceps femoris implies that the body’s ability to react in response to an impact is insufficient in this type of landing, which may increase the risk of injury. In addition, the median frequency of the muscles is elevated when landing on both feet simultaneously, reflecting increased muscle fatigue. This increased fatigue may make it more difficult for the body to maintain balance and postural control, especially during everyday activities that require quick reactions. In contrast, landing on both feet, fore and aft, is more likely to help elevate muscle activation levels, allowing the body to more effectively distribute the impact of the landing, thereby improving stability [35]. Therefore, this finding is an important guide for the choice of landing stance in daily life. Whether it is for sports or daily activities, the use of the anterior-posterior landing with both feet can better protect the body and reduce the potential risks caused by fatigue, thus improving the overall safety and stability.

This study has certain limitations that should be acknowledged. Firstly, the sample size of 26 participants may not be large enough to generalize the findings across a broader population. Future research could benefit from including a larger and more diverse sample to enhance the reliability of the results. Secondly, this study focused on a specific height of 120 cm for jumping, without comparing different jump heights. It would be valuable for future studies to investigate the injury risks associated with various heights, as this could provide a more comprehensive understanding of the biomechanical parameters involved in high-altitude landing maneuvers.

## 5. Conclusions

In this study, the effect of landing posture on injury risk at altitude was thoroughly investigated through the integrated application of advanced Xsens inertial sensors and surface electromyography, combined with AnyBody to establish a kinetic musculoskeletal model. The results showed that staggered landing (landing with front and back feet successively) can increase the muscle activation level and disperse the landing impact more efficiently, thus improving stability and reducing the potential injury risk due to fatigue. On the contrary, landing on both feet at the same time may increase joint loading and increase the risk of injury. Therefore, it is recommended that athletes and coaches prioritize the use of a staggered landing position between the anterior and posterior feet during training and daily activities to improve safety and stability, thereby enhancing training efficiency and competitive performance. This study provides theoretical support for further research in the field of sports biomechanics and provides important guidance for safety in sports training.

## Figures and Tables

**Table 1:** Comparison of kinematic variables between different landing positions (Unit: °).

Parameter	Staggered Foot Landing	Simultaneous Bilateral Landing	*p*
Hip flexion-extension	32.83 ± 2.24	43.12 ± 3.17	<0.001 *
Hip adduction-abduction	21.83 ± 1.04	22.12 ± 1.26	0.369
Knee flexion-extension	60.54 ± 3.02	83.73 ± 2.35	<0.001 *
Ankle dorsiflexion-plantarflexion	20.55 ± 2.19	21.62 ± 2.54	0.110
Ankle inversion	11.55 ± 0.29	15.62 ± 0.36	<0.001 *
Ankle eversion	4.57 ± 0.18	4.68 ± 0.23	0.061

* indicates that the difference is significant.

**Table 2:** Comparison of vertical joint forces between different landing positions (Unit: N/BM).

Parameter	Staggered Foot Landing	Simultaneous Bilateral Landing	*p*
L1–L2	2.05 ± 0.65	2.26 ± 0.07	0.113
L2–L3	2.39 ± 0.38	2.42 ± 0.34	0.765
L3–L4	3.56 ± 0.89	3.65 ± 0.66	0.680
L4–L5	6.15 ± 0.74	8.45 ± 1.07	<0.001 *
Hip joint	11.54 ± 1.65	14.56 ± 1.11	<0.001 *
Knee joint	14.24 ± 0.94	16.83 ± 1.56	<0.001 *
ankle joint	16.24 ± 1.11	19.78 ± 1.36	<0.001 *

* indicates that the difference is significant. L is for lumbar.

**Table 3:** Comparison of joint moments between different landing positions (Unit: Nm/BM).

Parameter	Staggered Foot Landing	Simultaneous Bilateral Landing	*p*
Hip adduction-abduction	1.14 ± 0.29	1.17 ± 0.26	0.696
Hip flexion-extension	0.72 ± 0.22	0.77 ± 0.14	0.333
Hip rotation	0.17 ± 0.02	0.16 ± 0.04	0.261
Knee flexion-extension	1.86 ± 0.12	2.51 ± 0.13	<0.001 *
Ankle dorsiflexion-plantarflexion	0.40 ± 0.04	0.41 ± 0.07	0.531
Ankle inversion-eversion	1.01 ± 0.17	1.55 ± 0.30	<0.001 *

* indicates that the difference is significant.

**Table 4:** Comparison of muscle forces simulated by AnyBody for different landing positions (Unit: N/BM).

Parameter	Staggered Foot Landing	Simultaneous Bilateral Landing	*p*
Gluteus maximus	1.04 ± 0.19	0.78 ± 0.28	<0.001 *
Iliopsoas	2.76 ± 0.14	2.01 ± 0.31	<0.001 *
Adductor magnus	1.53 ± 0.25	1.47 ± 0.11	0.270
Sartorius	0.19 ± 0.014	0.18 ± 0.07	0.481
Quadriceps femoris	4.71 ± 0.38	2.35 ± 0.75	<0.001 *
Biceps femoris	1.86 ± 0.24	1.05 ± 0.31	<0.001 *
Semimembranosus	5.84 ± 0.38	4.03 ± 0.58	<0.001 *
Tibialis anterior	0.54 ± 0.03	0.53 ± 0.10	0.628
Soleus	1.12 ± 0.28	1.15 ± 0.15	0.632

* indicates that the difference is significant.

**Table 5:** Comparison of integrated EMG and median frequency between different landing positions.

Metric		Staggered Foot Landing	Simultaneous Bilateral Landing	*p*
Integrated EMG (μV·s)	Gluteus maximus	125.76 ± 19.49	127.62 ± 17.68	0.720
	Quadriceps femoris	126.74 ± 18.66	120.16 ± 21.30	0.241
	Biceps femoris	71.95 ± 16.88	60.41 ± 13.03	0.008 *
	Gastrocnemius	74.97 ± 10.36	75.36 ± 11.22	0.896
	Tibialis anterior	58.47 ± 14.90	59.38 ± 12.36	0.811
Median Frequency (Hz)	Gluteus maximus	21.35 ± 3.99	21.29 ± 3.32	0.953
	Quadriceps femoris	16.07 ± 5.28	22.98 ± 6.69	<0.001 *
	Biceps femoris	25.76 ± 5.09	26.37 ± 3.22	0.608
	Gastrocnemius	14.09 ± 4.98	15.65 ± 6.70	0.345
	Tibialis anterior	15.77 ± 2.42	16.53 ± 2.73	0.293

* indicates that the difference is significant.

## Data Availability

All data are in the manuscript; please contact the corresponding author if you need anything else.
